# The epidemiological aspect of traffic injuries mortality in East Azerbaijan province, Iran

**Published:** 2019-08

**Authors:** Saeid Pour Doulati, Mohsen Pasha, Jabraeil Sharbafi, Fatemeh Anvari, Nayer Sadegpour, Leila Abdollahi

**Affiliations:** ^*a*^Center for Non-Communicable Diseases Control and Prevention, East Azerbaijan Province Health Center, Tabriz, Iran.; ^*b*^Alzahra Hospital, Tabriz Medical Sciences University, Tabriz, Iran.

## Abstract

**Background:**

Traffic injuries are the third leading cause of death in Iran, accounting for more than 16000 death in 2016 (death rate of 20.5 per 100000 population). It was the first leading cause of premature death and disability in Iran in 2003. East Azerbaijan province is ranked eighth among the 31 provinces in terms of the number of deaths caused by traffic injuries. Purpose: To determine the epidemiological aspects of fatal traffic injuries in East Azerbaijan province, Iran.

**Methods:**

This cross-sectional descriptive study included 758 traffic-related deaths in East Azerbaijan province between March 2016 and March 2017. The data source was the Ministry of health and medical education death registry. According to this registry, all deaths should be recorded in this system. In this study, demographic and epidemiological variables including age, sex, education, occupation, death place, and living area were collected. Data were analyzed using Excel 2013 software.

**Results:**

Out of 758 people died in traffic injuries, 604 (80%) were male and 154 (20%) were female. The age distribution of death indicated that 354 (57%) aged 20-59 died from road traffic injuries. 667 (53.2%) road traffic death happened in the road and public places, 386 (30.3%) deaths happened in hospitals and day clinics, and 210 (16.5%) cases were unknown. In terms of living area 503 (66%) were living in urban areas, 242 (32%) in rural areas and it was unknown for 13 (2%) cases. 144 (19%) cases had primary education, 113 (14.9%) secondary education (Guidance school), 125 (16.5%) tertiary education (high school), 116 (15.3%) were uneducated, and 78 (10.3%) had academic education. In respect of occupation In terms of occupation, most jobs respectively were related to free job (36.7%) , unknown (22.8%), housekeepers (12.5%), farmers (7.4%), students (6.7%), employees/laborers (6.5%), unemployed (5%), retired (4.1%), children (2.4%), and professional drivers (1.8%).

**Conclusions:**

Considering that the most road traffic deaths occurred in roads, paying more attention to roads and vehicles safety should be taken into account. The high rate of death in men aged 20-59 who mostly are family breadwinners indicates the necessity of the effective behavioral interventions for this group.

**Keywords:**

Road traffic injuries, Fatality, Road accident

